# A cross-sectional study of maternal perception of fetal movements and antenatal advice in a general pregnant population, using a qualitative framework

**DOI:** 10.1186/1471-2393-13-32

**Published:** 2013-02-05

**Authors:** Camille H Raynes-Greenow, Adrienne Gordon, Qiushuang Li, Jon A Hyett

**Affiliations:** 1 Sydney School of Public Health, University of Sydney, Sydney, NSW, 2006, Australia; 2 Department of Neonatal Medicine, Royal Prince Alfred Hospital, Sydney, NSW, Australia; 3 Discipline of Obstetrics, Gynaecology and Neonatology, University of Sydney, Sydney, NSW, Australia

**Keywords:** Pregnancy, Fetal movement, Qualitative analysis, Cross-sectional study

## Abstract

**Background:**

Maternal perception of fetal movements has been used as a measure of fetal well-being. Yet a Cochrane review does not recommend formal fetal movement counting compared to discretional fetal movement counting. There is some evidence that suggests that the quality of fetal movements can precede quantitative changes however there has been almost no assessment of how women describe movements and whether these descriptions may be useful in a clinical setting. Therefore we aimed to examine maternal perception of fetal movements using a qualitative framework.

**Methods:**

Using a cross-sectional design we identified women during routine antenatal care at a tertiary referral hospital, in Sydney, Australia. Eligible women were pregnant ≥ 28 weeks, carrying a single child, > 18 years old, and with sufficient English literacy to self-complete a questionnaire. Post-natally the medical records were reviewed and demographic, pregnancy and fetal outcome data were extracted. Text responses to questions regarding maternal descriptions of fetal movements throughout pregnancy, were analysed using thematic analysis in an explicit process.

**Results:**

156 women participated. There was a general pattern to fetal movement descriptions with increasing gestation, beginning with words such as “gentle”, to descriptions of “strong” and “limb” movements, and finally to “whole body” movements. Women perceived and described qualitative changes to fetal movements that changed throughout gestation. The majority (83%) reported that they were asked to assess fetal movements in an implicit qualitative method during their antenatal care. In contrast, only 16% regularly counted fetal movements and many described counting as confusing and reported that the advice they had received on counting differed.

**Conclusions:**

This is the first study to use qualitative analysis to identify that pregnant women perceive fetal movements and can describe them in a relatively homogenous way throughout pregnancy that follow a general pattern of fetal growth and development. These findings suggest that women’s perception of fetal wellbeing based on their own assessment of fetal movement is used in an ad hoc method in antenatal care by clinicians.

## Background

Regular fetal activity perceived by pregnant women has long been regarded as a sign of fetal wellbeing [1] and continues to be used by women and clinicians [2]. Decreased fetal movement has been associated with poor pregnancy outcomes including stillbirth [3]. Physiological studies of fetal activity have found associations between decreased fetal movement and poor perinatal outcome [4].

Maternal perception of decreased fetal movement has been reported in 15% of pregnancies during the third trimester [5] and around 50% of women perceive a gradual reduction of fetal movement days before intrauterine death [6-8]. Thus early detection of reduced fetal movement has been considered as an opportunity for fetal health screening. A systematic review listed formal fetal movement counting as a potential intervention for reducing stillbirths in low and middle-income countries [9].

Research has invested considerable efforts into evaluating interventions into maternal perception of fetal movement and has focussed on quantification of the movement specifically counting [10-13]. However, a Cochrane review [14] including over 71,000 women, comparing different methods of formal fetal movement counting, found equivocal results, with no advantages to formal fetal movement counting compared to discretional fetal movement counting.

There is some evidence that changes in the quality of fetal movement can precede quantitative changes [3,10,15] yet there has been almost no assessment of how women describe movements and whether these descriptions may be useful in a clinical setting. The paucity of studies on fetal activity patterns and maternal perception of this activity in normal pregnancies has been identified by the Royal College of Obstetrics and Gynaecology (RCOG) as contributing to the evidence gap and as an area requiring more research [16]. The current evidence includes a recent study of 40 women who used words such as “strong and powerful” to describe their baby’s movements in the two weeks before the birth of their live born infant [17], and a case–control study reported some qualitative differences in maternal perception between mothers of stillborn cases and live born controls [18].

The first aim of this study was to examine maternal perception of normal fetal movements in a general pregnant population using a qualitative framework, and second was to describe fetal movement advice in a routine antenatal care setting.

## Methods

### Study design and sample

This was a cross-sectional study conducted at a major metropolitan tertiary referral hospital in Sydney, Australia with ~ > 5000 deliveries per year. The population that it services is an inner-city urban, multicultural population, and the predominate ethnicities include Anglo-Celtic, South-East Asian and Middle Eastern. At the time of the study the hospital gave a series of information leaflets to pregnant women at the initial booking visit (between 12–19 weeks) encouraging women to “be aware of fetal movements”. There was no other routine practice regarding fetal movements. Eligible women were pregnant ≥ 28 weeks gestation, carrying a single child, over the age of 18, able to consent to participation and were sufficiently competent in English to self-complete the questionnaire. A sample size of 150 women was determined sufficient to explore the construct of fetal movement perception by gestational strata. We purposely recruited in three gestational strata, between 28–31, 32–36 and 37+ weeks gestation.

Ethics approval was obtained from the relevant Human Research Ethics Committee, Protocol no. X10-0318 and informed consent was obtained from all participants.

### Questionnaire design

The questionnaire was based on one we developed for the Sydney stillbirth case–control study [19,20]. The initial development included an iterative process of review and revision, with content experts, and was pilot tested in a convenience sample of pregnant women. There were no changes to the questions for this study, other than to convert the style from an interview style questionnaire to a self-complete questionnaire. Both fixed response and open ended questions were included and it took between 5 to 15 minutes to complete. Question topics included description of fetal movements; how fetal movements felt during early pregnancy and how the movements felt as the pregnancy progressed, whether movements developed a (time) pattern throughout the day or night, if there had been an unusual experience of fetal movements, and what information had been received from health professionals.

### Data collection

Women over 28 weeks gestation were identified from the booking sheets for each day of the study period and approached. After reading the patient information sheet and giving informed consent, each woman self-completed the questionnaire. Responses were de-identified with a study number allocated to each questionnaire. Post-natally the medical records were reviewed and demographic, pregnancy and fetal outcome data were extracted.

### Data analysis

Text responses to open-ended questions were analysed using thematic analysis [21], in a five stage process by two members of the team (CRG, QL): familiarisation with the data (reading and rereading transcripts), independently coding the transcripts using the study objectives and emergent themes, comparing codes between interviews and re-coding if necessary (a third researcher participated in this process (AG), developing a conceptual framework by clustering themes together into broader categories; and finally summarising and synthesising data. The final discussion between the researchers continued until there was a consensus of themes. For data presentation we selected quotations that represented or were typical of the experience of a number of participants or because they stood out as examples of an a typical experience. Quantitative data are presented using frequency tabulations.

## Results

### Recruitment and participant characteristics

Of the 166 eligible women in the antenatal clinics, 160 agreed to participate. Of the 6 women who declined participation, reasons included ‘cannot be bothered’, ‘were busy’ and ‘disliked forms’. Of the women who agreed to participate, 4 could not complete the questionnaire because they were called into their appointment. We therefore included 156 women, a response proportion of 94%; 50 at 28–31 weeks, 55 at 32–36 weeks and 51 at 37+ weeks gestation. We were able to extract data from 97% (151) of the medical records, two women did not wish their medical records to be collected and 3 records were missing. For these women their questionnaire data are included, and they are recorded as ‘not reported’ in Table 1. All pregnancies resulted in live births.

**Table 1:** Characteristics of participants

Maternal Characteristics N = 156*	**n**	**(%)**
Maternal age (years, mean, low - high)	32.6	(18–45)
Gestation at recruitment (weeks)		
28–31	50	(32)
32–36	55	(35)
> 37	51	(33)
Primary model of care		
Midwives clinic	104	(66.7)
GP shared care	35	(22.4)
Birth centre	4	(2.6)
not recorded**	13	(8.3)
Parity		
Primiparous	90	(57.7)
Multiparous	50	(32.0)
not recorded	12	(10.3)
Maternal BMI - kg/m^2^	mean 23.0	SD (± 4.6)
Smoking during pregnancy		
Yes	10	(7)
No	121	(80)
not recorded	20	(13)
Any alcohol during pregnancy		
Yes	8	(5)
No	116	(77)
not recorded	27	(18)
Gestational age at birth (weeks)	mean 39.3	high-low (35.6–41.6)
Birth weight (grams)	mean 3405	high-low (2400–4900)
Type of labour		
Spontaneous	76	(50)
Induced	49	(32)
No labour	26	(17)

*Not all data were available for all records.

** Not recorded data includes the 5 women whose data was not available.

The mean maternal age was 32 years. All women received care through public clinics as we did not recruit women through private clinicians. The majority were being cared for through a midwife-led clinic 104 (66.7%) and were primiparous 90 (57.6%). The mean body mass index (BMI) of participants was 23 kg/m^2^ (Table 1).

### How do fetal movements feel?

We asked women to describe how their baby’s movements felt in early pregnancy and as their pregnancy progressed (Table 2). Women’s description of their baby’s first movements could be categorised into three main themes, abstract interpretation, emotions and verbs describing human actions. Abstract interpretations included being very gentle;

“very soft movement, like a feather inside my belly” (ID042, 36wks).

“It was like a little puff of air from your mouth into my tummy, like a soft kiss. It was very gentle…” (ID084, 35wks).

**Table 2:** Thematic analysis of qualitative responses, by major themes, sub categories, and words

**Major theme**	**Sub-category**	**Words**
***“How did you baby’s first movement feel”***		
Abstract movements	Gentle	Gentle, soft, light, faint, hard to notice, feather
	Gas	Bubbles, wind, gas, air, bowel movement, stomach rub, gurgling, heartburn
	Flutter	Flutter, butterfly wing, ripple/waves, tingle, buzz, whoosh, itch, stomach spasm
Feelings of the mother	Positive	Amazing, wonderful, comforting, good, nice, peaceful, exciting, joy/happy
	Negative	discomfort, nauseating, pinch, irritating, creeping
	Neutral	strange, foreign, weird, surprise
Early actions	Limb	Push, kick, jolt, punch, stab/jab, tap, scratching, thump, knock, poke, flick
	Whole body	Push, twists, swirl, spin, roll, wriggle, throbbing, hiccups, pulsing
***“How have your baby’s movements felt as the pregnancy progressed?”***		
Changes	Increasing power	Harder, stronger, more intensity, more force, more vigorous
		Sharper shorter
		Uncomfortable, painful, hard to sleep, more aggressive
	Bigger movements	visible, identifiable features, more definite, “fighting for space” “trying to escape from belly” “felt all over”
	frequency/duration	more frequent, more active, peak in week 28
		Less sudden, sharp, sustained and constant
		increased repetitive sensation/hiccups, pulses
Action/Type of movements	Short and sharp	kicking, drums, punch, hit, strike, thump, nudge, poke, jab, tap, flick, jerk, twitch
	smooth	Bumps, squirming, stretching, rolling/change position, swipe, wave, churning, side to side,, twists, somersault, swoop, pushing, pressing
	complex	swimming, running

Some women described quick and repetitive movements such as “flutters” or “butterflies”, whereas others likened the feeling of the movements to “air” or “gas” or “bubbles popping”. One woman wrote;

“I thought the feeling was my mobile phone ringing in my handbag… I could feel the bubbles and buzz of movements. Amazing.” (ID120, 28wks)

Many mothers expressed their emotional response to feeling their baby’s first movements. Overwhelmingly, these emotions were positive; however a few women expressed negative feelings;

“was irritating me a lot because the baby keeps moving.” (ID110, 34wks)

Finally, mothers often described their baby’s movements as an action. Actions perceived in early pregnancy were “gentle kicks” and “hiccups”. As the pregnancy progressed, women’s descriptions fell into two main categories; changes in the type of action, and relative changes in the size, strength or frequency of movements. Action type descriptions included specific limb descriptions and the whole body. Limb movements were described as short and sharp, “punchy” while whole body movements were “smooth”. Some women described a transition in the type of movements, associated with fetal growth;

“Movement has gradually changed from fluttering to stronger kicks and pushes.” (ID067, 37wks),

“Early movements were sudden kicks and jerks. Later they became more sustained, rolling, churning sensations.” (ID026, 39wks).

Although many women described this transition in fetal movements the exact timing was not apparent when comparing the text responses from different gestational groups as there was considerable overlap.

Simultaneously, women further reported a relative change in the size, strength and frequency of movements. Some women described that movements were becoming bigger, more visible and were identifiable as feet, or elbows, hands. Others described that space was an issue and that the baby was “fighting for space” (ID128, 29wks) or “trying to escape from my belly” (ID055, 39wks).

We asked women if there had been a change in the strength of fetal movement in the last 2 weeks, most of the women, regardless of gestation, reported that there had been a noticeable increase in the strength of movements 107 (70%). Almost all of these women expressed that movements became harder and stronger with some describing; sharpness, discomfort and pain. Only 2 reported a decrease in strength (38, 39 weeks gestation) however one qualified this with the comment; “(it) feels like less strength because of less room.” (ID143, 38wks).

When questioned whether they had experienced a change in the number of movements in the last 2 weeks, 86 (56%) reported they had noticed a change in the number of movements. Of these women, the majority described an increase in the frequency of movement with more regular periods of activity. However, some women, all between 32 and 41 weeks gestation, expressed movements were becoming more sustained, slower and occurring less often, many reported “squirming”. “…stronger, less kicks more squirming.” (ID 121, 37 weeks). Women justified the slower movements as caused by “space constraints” (due to increase in size of the baby) but still described an increased strength of movements. No women described this change in the frequency of movement as unusual.

### Unusual movements

When asked whether they had noticed anything unusual with their baby’s movements, 21 (14%) reported that they had experienced something unusual (12 of whom were primiparous), another 11 (7%) were unsure. These unusual movements included; the “absence of movements for 3 days” (ID021, 36wks), “a shudder” (ID121, 37wks), a “seizure type movement around the 20 week mark… (which felt) extremely rapid and jittery” (ID131, 39wks) and “a dull, constant yet high level pain around 30 weeks lasting 30 mins” (ID122, 33wks). Other movements described as unusual included pain, hiccups and occasionally movements not occurring at a “usual time”.

We further asked women what (if anything) they had done following the perception of unusual fetal movements. Nine women reported that they had done nothing. The responses of the remaining (12) were categorised into two themes; immediate or delayed response. Those (8) who acted immediately reported; drinking cold water, lying down, putting their hand on their abdomen, having a warm bath, eating, taking paracetamol and monitoring the movements. Only one woman reported immediately calling her care-provider for advice. Those who delayed any action described their action as “waiting” and being “worried” or they tried to rationalise the unusual movement. For example “…the baby must have found a new trick movement” (ID056, 31wks). Of the women who reported delayed actions, five women reported the unusual movement at their next antenatal appointment. Responses are not mutually exclusive.

### Timing of movements

**Figure 1:**
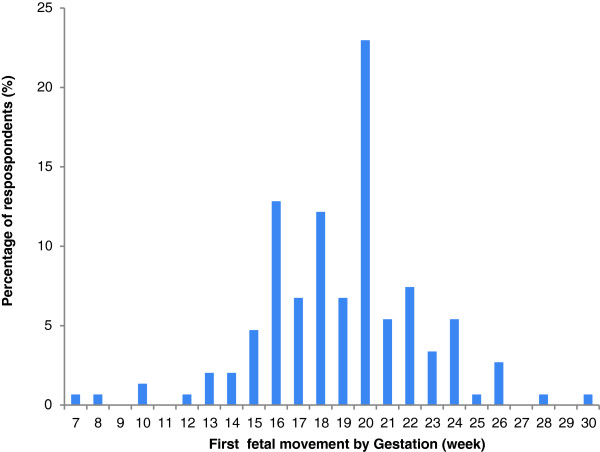
Maternal perception of first fetal movements by gestation (week).

The earliest reported perception of fetal movements was 7 weeks and the latest was 30 weeks (mean 19 weeks, Figure 1). Over a quarter of women experienced their first movement after 20 weeks (26%), and half of those after 22 weeks.

**Figure 2:**
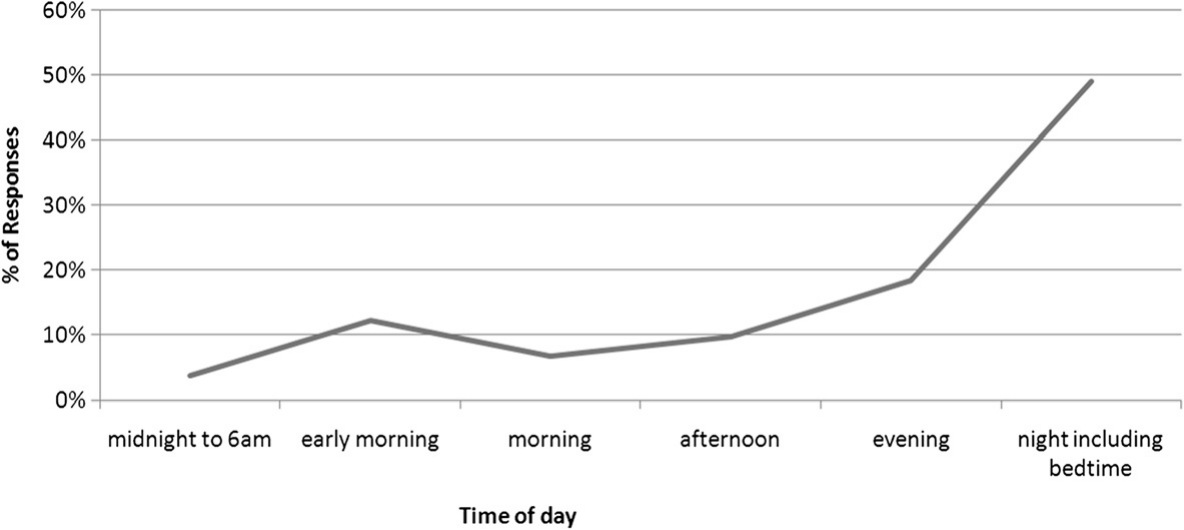
“What time of the day or night do you feel your baby move the most?”.

Responses to the question “When do you feel the most fetal movements?” were categorised into three themes either; ‘a time of day’, ‘with food consumption’ or ‘with an activity’. Women who were not able to identify a particular time often reported “all the time” (ID059, 38wks) or “regularly throughout the day” (ID143, 38wks). Time responses were further coded into 6 periods of the day, including ‘predawn’ (before 6am), ‘early morning’ (6–8am), ‘morning’ (8–12 noon), ‘afternoon’ (12–6pm), ‘evening’ (6–8pm) and ‘night time including bedtime’ (8–12 midnight). The results show an upward trend of fetal movement perception throughout the day peaking during the evening (Figure 2).

Increased perception of movement was also associated with certain maternal body positions, including sitting, lying down or resting for 26 (17%), specifically these included “lying down on the side” (ID114, 30wks) or sitting down with “feet up” (ID152, 29wks). Only two reported more movements during exercise. Food consumption, or feeling hungry, was associated with more movement perception 19 (12%). Of these, most were associated with the evening meal. Coffee, sugary beverages and or cold water were all reported to stimulate fetal activity.

### Fetal movement discussion during routine antenatal care

Although most women (83%) described being asked about fetal movements during their routine antenatal appointments, 12% could not recall any fetal movement questions. Antenatal care providers typically asked questions from one of four implicit themes; 1: Any movements; “Is the baby moving?” (ID018, 33wks), 2: Regular and frequent movements, 3: Any change from usual movements, and 4: Are the movements normal; “Are you happy with number of movements?” (ID121, 37wks). Only 5 women reported being asked specifically about the number of movements “Do (you) feel at least 10–12 movements in 24 hours” (ID105, 31wks). Some women reported that questions regarding fetal movements were insufficient.

“nothing specific … doctor just said as long as baby’s moving, it’s ok. Though I would like to have more explanation” (ID146, 35wks).

Although not all women reported discussing their fetal movements during their routine care almost two thirds had received advice about fetal movements 106 (69%). Advice provided to women were coded under three themes; ‘normal number of movements’, ‘normal pattern of movements’ and ‘action to take if women were concerned’. Responses concerning the normal number of movements varied greatly, including 10 every 2 hours, 10 every 12 hours, 10–20 every 24 hours, every 3 hours and at least once per day. Women expressed their confusion about how to count:

“… (the information was) conflicting as to how many times he should move and whether I should count every kick or count every session of movements” (ID084, 35wks).

Other advice focused more on the presence of movement rather than quantity

“(care-provider said) do not worry too much and not to count movements, just ensure there is movement everyday” (ID098, 36wks).

Advice regarding the normal pattern of movements included what movements should feel like, how frequent and how it should progress:

“(you will) feel less kicking as the baby gets bigger (and) has less room to move” (ID097, 37wks).

“although the movements may slow down you should feel movements right up until you go into labour” (ID107, 39wks).

Some advice focused on the individuality of fetal movements;

“Monitor what is normal for you personally and to note any deviations from said behaviour” (ID132, 41wks).

Of the women who received fetal movement advice, most (77%) recalled receiving advice regarding what to do if they were concerned. This advice included taking a “timeout” (lie down), “monitoring and recording to see if it is above the minimum”, “try to stimulate movement” (drinking a cold or a hot drink) and “telephone the labour ward”. Three women reported that they were advised to seek help immediately if they noticed decreased fetal movements.

Medical professionals (doctor and or midwife) were the most frequent source of fetal movement advice (37%), however they were not the only source of advice reported and not the first source. Pregnancy books (19%), friends (15%), and the internet (13%) were commonly the first source of advice. Antenatal classes (11%) and other (4%) including family, pamphlets and yoga teacher were also used, another 8% did not answer. The internet and or books were reported as a good source of information as they were easily and quickly accessible, “… if I ever have questions or worries I usually ‘Google it’ or look up a book.”(ID116, 37wks).

Formal fetal movement counting was reported by 16% of women however we did not ask specifically what method they had used.

## Discussion

In this study, we found that women were able to recognise qualitative changes in fetal movements throughout gestation. The data also revealed an overall identifiable pattern associated with pregnancy, beginning with descriptions of ‘gentle’ movements, ‘bubbles’ and ‘butterflies’. Towards mid pregnancy these were described as ‘bigger’ and ‘sharper’ movements which included association with limb movements. Finally women towards the end of pregnancy, generally described fetal movements as ‘smooth’, ‘sustained rolling sensations’. These movement descriptions coincide with fetal development at this gestation which includes improved fetal coordination and limb control [3,22] and increased fetal size [23]. This overall pattern is supported by physiological data of fetal motor development understanding [24,25]. Importantly, only two women interpreted this change as reduced frequency of fetal movement but still described an increase in strength of movements.

Most women used words that described an increase in strength of movements throughout their pregnancy regardless of gestation. From 32 to 41 weeks gestation, some women reported less movements but all described increasing strength. Our findings suggest that pregnant women are aware of the quantitative movement changes and are aware of increasing strength. The mean gestation for women to perceive first movements was 19 weeks, in accordance with previous research [16,23]. However, our results also found that over a quarter of women recognised their first movement after 20 weeks, and this may be useful information for both clinicians and pregnant women. Earliest detection of fetal movement was 7 weeks, which concurs with a small ultrasound study of women who reported that there are “just discernible movements” from 7 weeks (10).

Perception of movement increased as the day progressed, peaking during the night which is consistent with previous research [23,24]. We speculate that this may partly be a function of women’s attention, as many women reported that they noticed increased movement in the early morning when they are likely to be in bed when other distractions are likely to be minimised.

Importantly few women felt the need to seek advice immediately when they noticed an unusual fetal movement. Of the women concerned about unusual fetal movements, only one sought help within 24 hours. The remaining who told their care-provider, did so at the next antenatal appointment which (most likely) was past a critical time point for intervention. Previous research has shown that around 50% of women affected by unexplained stillbirth and observing an absence of fetal movement waited more than 24 hours before they contacted their health professional [7,26]. Guidelines recommend that women who are concerned about reduced fetal movements should not wait until the next day for assessment [16], again women in our study did not act in this way, although we did not collect data to measure if they had been instructed to do this.

Overall, routine antenatal care in regards to fetal movement was varied and inconsistent. This may be a reflection of several factors some of which are specific to this setting, including the lack of a policy on fetal movement monitoring at the time of this study, (there is now a policy) there is a now a current policy [27]. More generally it may be a function of the overall uncertainty regarding the evidence surrounding fetal movement monitoring, the lack of definitions of normal fetal movements or the definition of abnormal movements for identifying fetal compromise [26,28]. Although some women reported being unsatisfied with the general nature of the fetal movement questioning during their care, this may actually be the most appropriate type of questioning, as it aims to assess woman’s assessment of her baby’s movement [16].

Caregiver advice on the normal number of fetal movements varied greatly. Most women could not confidently recall the number or the time frame that fetal movements should be counted. Very many “Kick counting” methods exist and are used in antenatal settings around the world, however the evidence is unsupportive of routine use [14]. Almost all women reported being asked about fetal movements in their routine care, most of this questioning was centred on the woman’s qualitative assessment. This study provides evidence that supports the value of women’s perception of fetal movements to clinicians.

Strengths of our study include the large sample size of qualitative data. We developed an explicit analysis process (Table 2), and used multiple coders for research triangulation which adds validity to the findings [29]. The limitations of our study include lack of data regarding placental position or amniotic fluid volume, both of which have been shown to affect maternal perception of fetal movement [30]. The generalisability of the coding frame may be limited to similar cultural and language populations. Although there is no reason that the types of movements will differ in different cultures the words used to describe them may and thus these words would need to be tested for cultural specificity.

This work is the first step towards defining normal fetal movement using a qualitative framework, work such as this may help identify ‘alert words’ that women and care-providers could use in a clinical setting to screen for babies at risk of demise.

## Conclusion

Our results show that fetal movement during pregnancy follows a general pattern, and that women can perceive and describe these changes without any instruction. Although further work is required, maternal qualitative assessment of fetal movement appears to be included in routine care and requires no maternal training and thus offers a promising alternative to quantitative assessment.

## Competing interests

We do not have any interests to disclose.

## Authors’ contributions

CRG designed the study, supervised data collection. She also conducted data analysis, and was primarily responsible for data interpretation. QL collected the data, and analysed the data, assisted with data interpretation, and wrote the first draft of the manuscript. AG co-designed the study, contributed to the analysis, data interpretation and manuscript preparation. JH provided access and support for the study in the antenatal clinics, assisted with data interpretation and manuscript writing. All authors read and approved the final manuscript.

## Pre-publication history

The pre-publication history for this paper can be accessed here:

http://www.biomedcentral.com/1471-2393/13/32/prepub
